# One-Carbon Metabolic Factors and Risk of Renal Cell Cancer: A Meta-Analysis

**DOI:** 10.1371/journal.pone.0141762

**Published:** 2015-10-29

**Authors:** Bijing Mao, Yafei Li, Zhimin Zhang, Chuan Chen, Yuanyuan Chen, Chenchen Ding, Lin Lei, Jian Li, Mei Jiang, Dong Wang, Ge Wang

**Affiliations:** 1 Cancer Center, Institute of Surgical Research, Daping Hospital, Third Military Medical University, Chongqing, 400042, China; 2 Department of Epidemiology, College of Preventive Medicine, Third Military Medical University, Chongqing, 400038, China; Duke Cancer Institute, UNITED STATES

## Abstract

**Background:**

Nutrients related to one-carbon metabolism were previously shown to be significantly associated with the risk of cancer. The aim of this meta-analysis was to evaluate potential relationships between one-carbon metabolic factors and renal cell cancer (RCC) risk.

**Methods:**

PubMed, EMBASE, and Cochrane Library databases were searched through March 2015 for observational studies of quantitative RCC risk estimates in relation to one-carbon metabolic factors. The relative risks (RRs) with 95% confidence intervals (CIs) measured the relationship between one-carbon metabolic factors and RCC risk using a random-effects model.

**Results:**

Of the 463 citations and abstracts identified by database search, seven cohorts from five observational studies reported data on 133,995 individuals, and included 2,441 RCC cases. Comparing the highest with the lowest category, the pooled RRs of RCC were 0.72 (95%CI: 0.52–1.00; P = 0.048) for vitamin B_12_. In addition, an increase in folic acid supplementation of 100 μg/day was associated with a 3% lower risk of RCC (RR, 0.97; 95%CI: 0.93–1.00; P = 0.048). Similarly, an increase of 5 nmol/L of vitamin B_2_ was associated with a reduced risk of RCC 0.94 (95%CI: 0.89–1.00; P = 0.045). Sensitivity analyses suggested that a higher serum vitamin B_6_ might contribute to a reduced risk of RCC (RR, 0.83; 95%CI: 0.77–0.89; P < 0.001).

**Conclusions:**

Higher levels of serum vitamin B2, B6, B12, and folic acid supplementation lowered the risk of RCC among the study participants.

## Introduction

Renal cell cancer (RCC) is diagnosed in more than 120,000 patients in the USA and Europe, annually, resulting in nearly 60,000 deaths [1]. A third of patients with RCC are diagnosed in stage IV, with a 5-year survival rate of 15% approximately [2, 3]. Therefore, more effective preventive strategies to reduce the risk of RCC are needed. Recent studies have shown that several lifestyle factors such as high physical activity, alcohol, and intake of fruits and vegetables are associated with a lower incidence of RCC [4–11]. B vitamins are the main coenzyme precursors involved in the transfer of one-carbon groups and are essential for DNA methylation and DNA repair mechanisms [12]. Therefore, B vitamins have been linked with the risk of cancer [13]. Several meta-analyses [14–18] have evaluated the relationship between one-carbon metabolism and multiple cancers, but the relationship between one-carbon metabolic factors and the risk of RCC is not established.

Previous meta-analysis [9] indicated that protein or fat intake including red meat, poultry, and seafood might not be associated with the risk of RCC. Further, the dietary intake of fruits and vegetables has been closely related to the risk of gastric [19], prostate [20], colorectal [21], ovarian [22], and breast cancer [23]. Finally, another important study [24] suggested that consumption of cruciferous vegetables may be associated with reduced RCC risk. Among the supplemental nutrient subtypes, one-carbon metabolic factors may inhibit carcinogenesis and reduce the risk of RCC. However, data correlating one-carbon metabolism and subsequent incidence of RCC is limited.

Although a series of studies have evaluated the association between one-carbon metabolic factors and RCC risk, the results are controversial or inconclusive. Results of the present meta-analysis elucidate the relationship between one-carbon metabolism and the risk of RCC.

## Methods

### Data Sources, Search Strategy, and Selection Criteria

PubMed, EMBASE, and the Cochrane library were searched for articles published up to March 2015, using the search terms "renal cell carcinoma" OR "renal cell cancer" and "one-carbon metabolism biomarkers" or "folate" or “folic acid” or "vitamin B6" or “pyridoxine” or “cobalamin” or "vitamin B12" or "cysteine" or "riboflavin" or “thiamine” or "homocysteine". The search was limited to articles that were published in English. We also manually searched reference lists from all the relevant original research and review articles to identify additional potentially eligible studies. The literature search was performed in duplicate by two independent reviewers.

Inclusion criteria were: (1) observational studies investigating the relationship between one-carbon metabolism and the risk of RCC; and (2) those specifying the number of participants in each category of one-carbon metabolic factors. For studies without adequate data, we contacted the authors or searched the articles that reported a similar database. Studies without the necessary data were excluded.

### Data Collection and Quality Assessment

Data extraction and assessment were conducted independently by two authors. Publication information (i.e., first author’s name, and publication year), characteristics of the studies (i.e., country, study design, study quality, and adjusted factors), characteristics of participants (i.e., sample size, mean age, gender, educational background, body mass index [BMI], smoking, alcohol consumption, and history of hypertension), and the number of cases and participants in each category were extracted. Disagreement was resolved by consensus with a third reviewer.

Two reviewers independently evaluated the quality of the studies using the Newcastle—Ottawa Scale (NOS) (S1 Table) [25]. The NOS assessment is based on essential points of an observational study, i.e., selection (4 scores), comparability (2 scores), and outcome (3 scores). The three-point questionnaire produced a total score that ranged from 0 (the worst) to 9 (the best). In cases of a disagreement, a consensus was reached after a group discussion.

### Statistical Analysis

Effect estimate (RR, OR, or HR) and its 95% confidence interval (CI) were used to examine the relationship between one-carbon metabolic factors and the risk of RCC. Further, the risk estimates with maximal adjustment for potential confounders were used. Risk ratios (RRs) combined with the random-effects model were used as the summary statistic [26, 27].

The RRs were significant when the 95% CI did not include 1.00. First, the random-effects model was used to calculate summary RRs and 95% CIs for the high versus low one-carbon metabolic factors [27]. Second, category-specific risk estimates were transformed into estimates of the risk ratio (RR), which were associated with an increase in the level of one-carbon metabolic factors using the generalized least-squares method for trend estimation [28, 29]. The summary RRs for an increase in the level of one-carbon metabolic factors were calculated using random-effects meta-analysis [27]. Statistical heterogeneity among studies was evaluated using Q and I-square statistics, and P values < 0.10 indicated significant heterogeneity [30, 31]. Sensitivity analysis was used to explore potential sources of heterogeneity and to evaluate the influence of the included individual studies in our meta-analysis [32]. In the plan stage, subgroup analyses were used to explore the relationship between one-carbon metabolic factors, and the incidence of RCC risk in specific sub-populations. However, subgroup analyses were not conducted under conditions involving small number of trials.

In the planning stage, potential publication bias was evaluated by Egger [33] and Begg [34] tests. However, few studies reported the relationship between several one-carbon metabolic factors and the risk of RCC. All P values were two-sided and alpha values of P < 0.05 were considered statistically significant for all included studies. Statistical analyses were performed with STATA software (v. 12.0; Stata Corporation, College Station, TX, USA).

## Results

### Literature Search

**Fig 1:**
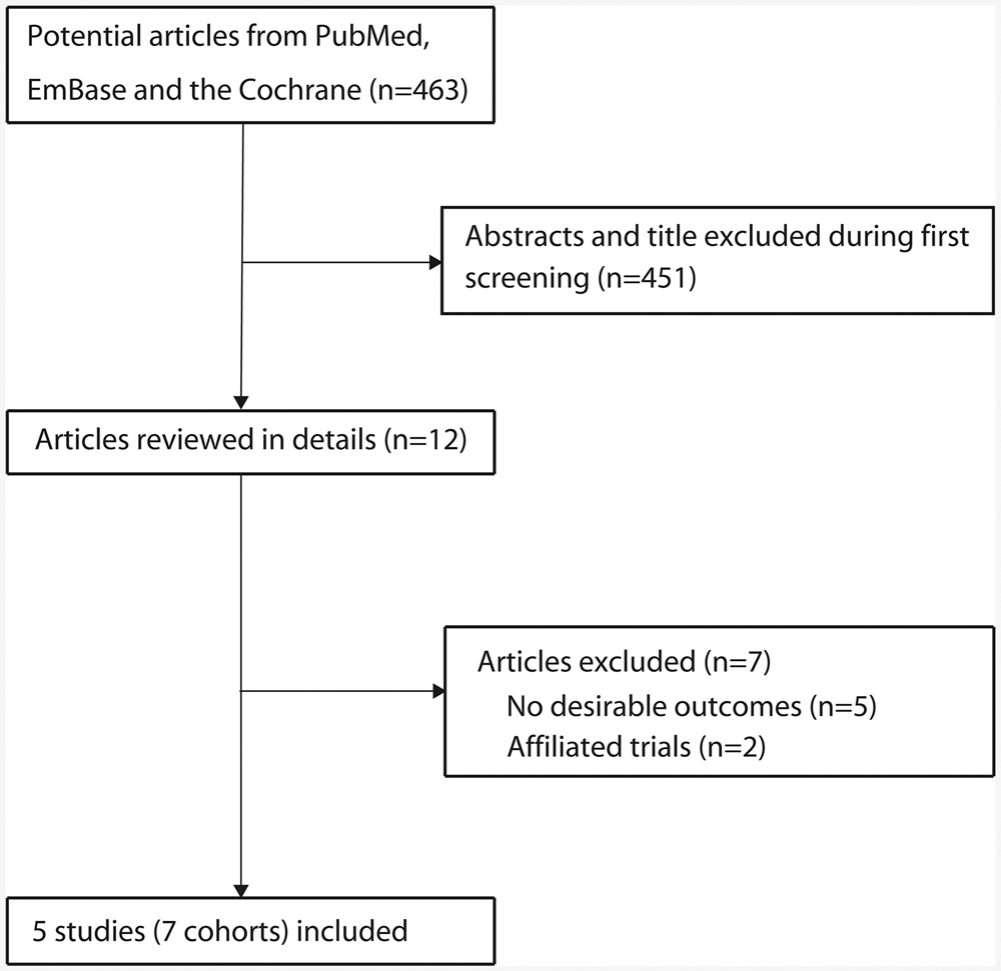
Flow diagram outlining the literature search and study selection process.

The primary search produced 463 records. After scanning titles and abstracts, 451 irrelevant articles were excluded. Twelve full-text articles were reviewed, and finally five studies [35–39] with seven cohorts were included in this meta-analysis (Fig 1). A manual search of the reference lists within these studies did not yield any new eligible studies. The general characteristics of the included studies and participants are presented in Table 1.

**Table 1:** Baseline characteristic of studies included in the systematic review and meta-analysis.

Variable	IMRCC [35]		NLCS [36]		EPIC [37]		ATBC [38]		NHS [39]		HPFS [39]	
	Cases	Non-cases	Cases	Non-cases	Cases	Non-cases	Cases	Non-cases	Cases	Non-cases	Cases	Non-cases
Country	Italy		Netherlands		10 countries in Europe		Finland		USA		USA	
Study design	Case control		Case cohort		Case control		Nested case control		Cohort		Cohort	
Assessment of exposure	FFQ		FFQ		Plasma samples		Plasma samples		FFQ		FFQ	
Study quality	7		8		9		8		8		8	
Sample size	767	1534	314	4438	556	556	224	224	225	76983	211	47675
Mean age (years)	62.0	62.0	61.9	61.4	56.9	56.9	57.0	57.0	61.0	61.0	62.0	62.0
Sex (percentage male)	64.4	64.4	65.9	49.4	56.0	56.0	100	100	0	0	100	100
Education (< 7 years)	48.5	55.3	-	-	41.0	37.0	79.5	78.6	-	-	-	-
Education (7–11 years)	27.6	29.8	-	-	22.0	25.0	-	-	-	-	-	-
Education (> 11 years)	23.9	14.9	-	-	37.0	39.0	-	-	-	-	-	-
BMI (< 25)	36.8	36.7	Mean: 25.5	Mean: 25.0	32.0	40.0	Mean: 26.7	Mean: 25.9	-	-	-	-
BMI (25–30)	45.4	49.1			45.0	43.0			-	-	-	-
BMI (> 30)	17.8	14.2			23.0	16.0			-	-	-	-
Smoking (never)	41.1	41.7	24.8	35.8	41.0	44.0	-	-	-	-	-	-
Smoking (current)	30.8	30.4	36.3	28.3	30.0	23.0	-	-	-	-	-	-
Smoking (ex-smokers)	28.1	27.9	38.9	35.9	29.0	32.0	-	-	-	-	-	-
Alcohol (never)	17.1	15.1	-	-	7.0	4.0	Mean: 7.4	Mean: 11.2	Mean: 6.0	Mean: 6.0	Mean: 11.0	Mean: 11.0
Alcohol (current)	74.7	77.5	-	-	92.0	95.0						
Alcohol (ex-drinkers)	8.2	7.4	-	-	2.0	1.0						
History of hypertension (yes)					35.0	25.0	-	-	37.0	37.0	32.0	32.0
History of hypertension (no)					50.0	59.0	-	-	63.0	63.0	68.0	68.0
Adjusted factors	Period of interview, education, BMI, smoking, alcohol intake and family history of kidney cancer		Age, sex, smoking, BMI and history of hypertension		Waist-to-hip ratio, hypertension, educational attainment, smoking status, plasma cotinine, alcohol intake at recruitment and alcohol intake.		Age, BMI and smoking; folate additionally adjusted for protein and fat; vitamin B6, riboflavin and homocysteine additionally adjusted for serum folate; vitamin B12 additionally adjusted for protein, leisure-time physical activity and serum folate.		Age, smoking status, BMI, history of hypertension, history of diabetes, physical activity, fruit and vegetable intake, alcohol intake, and parity		Age, smoking status, BMI, history of hypertension, history of diabetes, physical activity, fruit and vegetable intake, and alcohol intake	

### Study Characteristics

Three of the included studies were case studies [35, 37], two were cohorts [39], one was a nested case control study [38], and the remaining study was a case cohort [36]. These studies were published between 2006 and 2014, which comprised 133,995 individuals, and contained 2,441 RCC cases. Four cohorts were conducted in Europe [35–38], two were performed in the U.S. [39], and the remaining one was carried out in Australia [37]. IMRCC [35] and EPIC cohorts [37] reported education, BMI, and alcohol intake status. Similarly, three cohorts reported a history of smoking [35–37] and hypertension status [37, 39]. The quality of a study was evaluated using NOS, and one cohort scoring 9 [37], four cohorts scoring 8 [36, 38, 39], and the remaining two cohorts scoring 7 [35, 37].

### High versus Low One-Carbon Metabolic Factors

**Fig 2:**
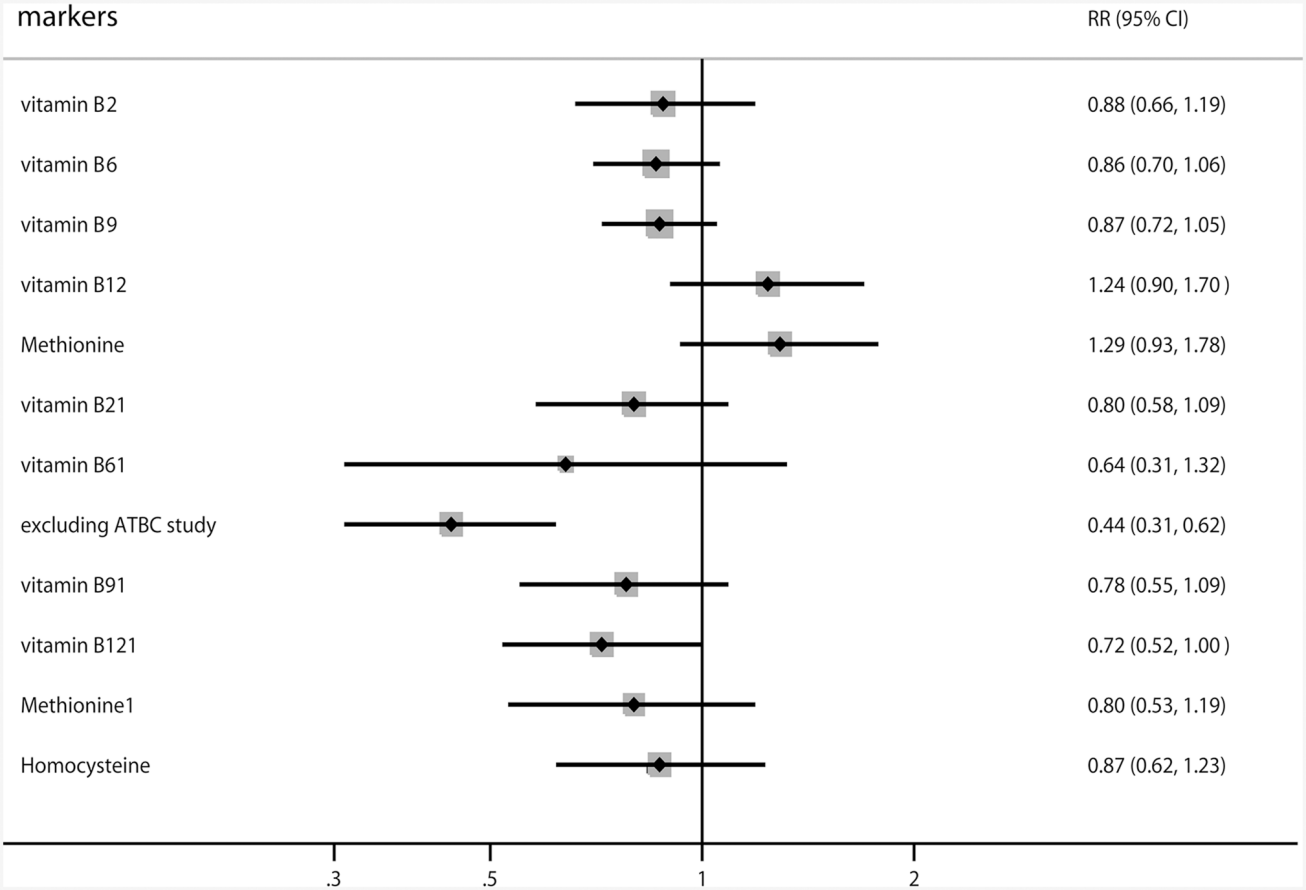
Summary of the calculated relative risks associated with high versus low one-carbon metabolic factors and renal cell cancer.

Fig 2 shows the RRs within the meta-analyses according to high versus low one-carbon metabolic factor levels. The summary RRs were 0.88 (95% CI: 0.66–1.19; P = 0.395) for vitamin B_2_ supplementation, 0.86 (95% CI: 0.70–1.06; P = 0.167) for vitamin B_6_ supplementation, 0.87 (95% CI: 0.72–1.05; P = 0.143) for folic acid supplementation, 1.24 (95% CI: 0.90–1.70; P = 0.194) for vitamin B_12_ supplementation, and 1.29 (95% CI: 0.93–1.78; P = 0.123) for methionine. Similarly, no significant associations were seen among plasma vitamin B_2_, plasma vitamin B_6_, plasma folate, plasma methionine, and plasma homocysteine levels. Further, compared with the lowest plasma category of vitamin B_12_, the pooled RR for RCC was 0.72 (95% CI: 0.52–1.00; P = 0.048). Finally, according to a sensitivity analysis, the highest category of plasma vitamin B_6_ was associated with a reduced risk of RCC (RR, 0.44; 95% CI: 0.31–0.62; P < 0.001) when excluding the ATBC study [38], which specifically included male participants and were aligned to a nested case control design.

### Dose-Response Analysis

**Fig 3:**
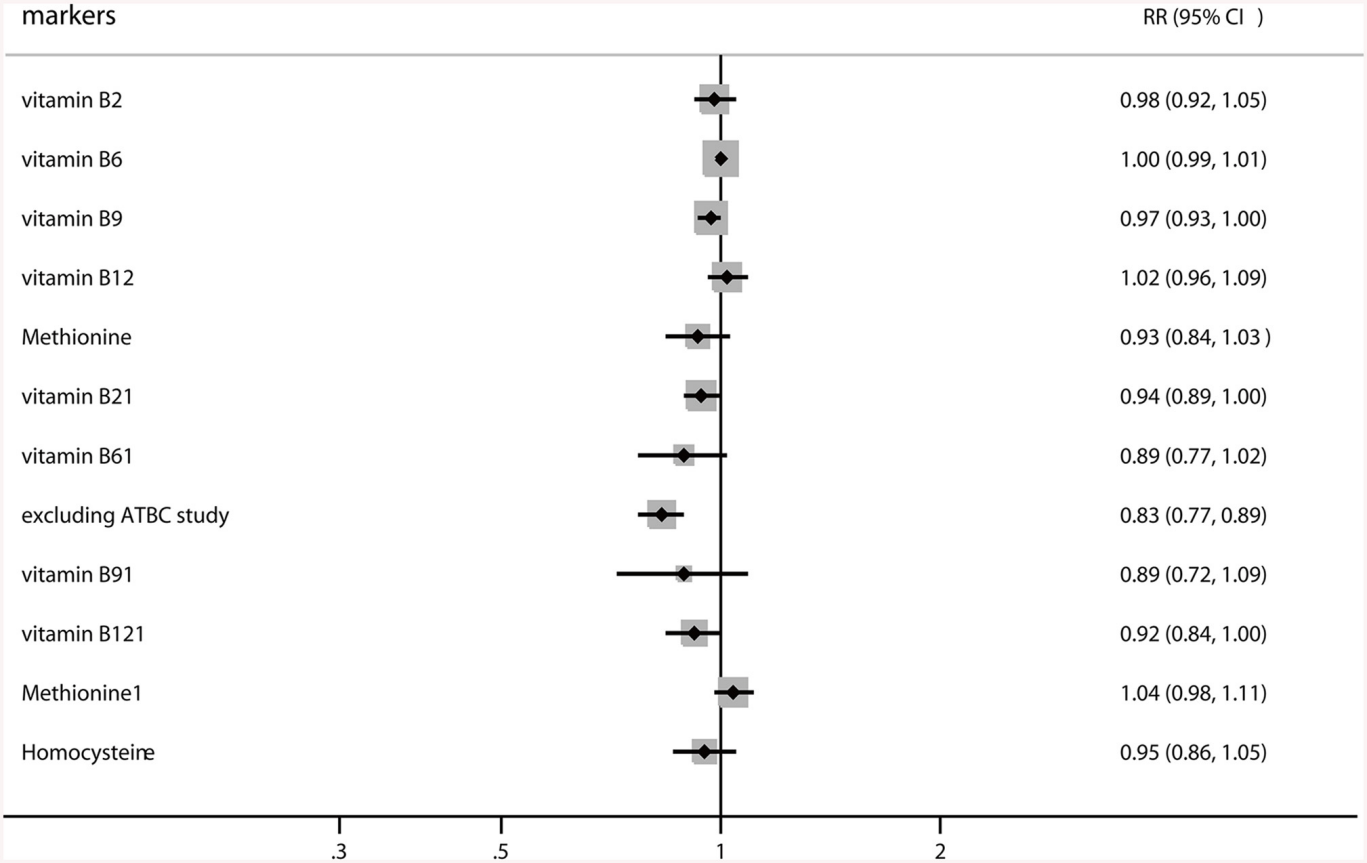
Summary of the dose-response meta-analysis of studies correlating increased one-carbon metabolism with subsequent risk of renal cell cancer.

The findings of the dose-response meta-analysis suggested a significant association between increase (100 μg/day) in folic acid supplementation and the risk of RCC (RR, 0.97; 95% CI: 0.93–1.00; P = 0.048). Furthermore, the summary RR of RCC for an increase in plasma vitamin B_2_ levels per 5 nmol/L was 0.94 (95% CI: 0.89–1.00; P = 0.045). In addition, the sensitivity analysis indicated that an increase in vitamin B_6_ by 15 nmol/L was associated with a reduced risk of RCC (RR, 0.83; 95% CI: 0.77–0.89; P<0.001) [38]. Finally, no significant associations were found between one-carbon metabolic factor increments and the risk of RCC (Fig 3).

## Discussion

Previous observational studies [35–39] correlating one-carbon metabolic factors with the risk of RCC have been inconclusive. EPIC and MCSS studies [37] found a decreased risk of RCC with high plasma vitamin B_6_ or vitamin B_12_. Several other studies failed to find any significant association with RCC, although most of the observed RRs were below unity [35, 36, 38, 39]. Further, the cut-off value of each category for one-carbon metabolism differed among studies. Finally, the incidence of RCC was lower than the expected value in individual studies, and always required broad confidence intervals, i.e., values exhibited no statistically significant differences. We therefore performed a comprehensive, quantitative meta-analysis to evaluate any potential relationship between one-carbon metabolic factors and the incidence of RCC risk.

This meta-analysis including published observational studies explored the potential correlations between one-carbon metabolic factors and the incidence of RCC. We found a statistically significant inverse association between folic acid supplementation, plasma vitamin B_2_ levels and the risk of RCC. Further, analyses of high versus low one-carbon metabolic factors indicated that plasma vitamin B_12_ was associated with a reduced risk of RCC. Finally, according to sensitivity analysis, vitamin B_6_ might play an important role in the risk of RCC.

Most of our findings were consistent with a recently published case control study that was conducted in ten European countries [37]. This study included 556 cases and 556 control subjects, which suggested that participants with higher plasma concentrations of vitamin B6 were associated with a lower risk of RCC. However, on the contrary, the other plasma biomarkers did not display any significant association with the incidence of RCC. In addition, a replication study that was conducted in Australia [37] suggested that the high plasma vitamin B_6_ levels were associated with a reduced risk of RCC (OR, 0.47; 95% CI: 0.23–0.99). Further, we used the generalized least-squares method for trend estimation and found that an increase in plasma vitamin B_6_ levels per 15 nmol/L, might be a protective factor for RCC. Finally, an ATBC study [38] indicated that participants with the lowest serum folate levels (≤ 6.64 nmol/L) had a 68% increase in the risk of RCC (OR, 1.68; 95% CI: 1.06–2.65) and a 22% increase in the risk of RCC per 100 μg, which was comparable to those with higher serum folate levels. Variables including study design, gender and source populations might play an important role in these associations. The ATBC study [38] included participants within a homogeneous population for randomization; however, EPIC and MCCS [37] were population-based observational studies. Furthermore, the biochemical measurements of vitamin B_6_ were performed with different methodologies. For example, the ATBC study [38] used tyrosine decarboxylase assay, and the EPIC and MCCS studies used chromatography/tandem mass spectrometry. Finally, we also conducted a sensitivity analysis excluding the ATBC study, which concluded that higher serum vitamin B_6_ was associated with a lower RCC risk.

Our current study also indicated that an inverse association remained statistically significant for folic acid supplementation (RR, 0.97; 95% CI: 0.93–1.00; P = 0.048). This result was consistent with IMRCC [35], which suggested that an increase in folic acid supplementation by 100 μg per day was associated with a reduced risk of RCC (RR, 0.94; 95% CI: 0.88–1.00). The other nutrients related to one-carbon metabolic supplementation were not associated with RCC. Studies on folic acid supplementation found that a higher intake was related to a reduced risk of oral, pharyngeal [40], breast [15], bladder [16], esophageal and pancreatic cancer [41]. However, the relationship between folic acid supplementation and the risk of RCC was unknown. Due to limited evidence supporting this association, and multiple nutrients related to one-carbon metabolism as demonstrated in this analysis, we conclude that the inverse association with folic acid, might be due to chance.

Our current meta-analysis has several strengths. First, the large sample size allowed us to quantitatively assess the association between one-carbon metabolic factors and the risk of RCC. Thus, our findings were potentially more robust than those of any individual study. Second, the dose-response analysis included a wide range of one-carbon metabolic factors, which allowed an accurate assessment of the relationship between per unit increments of one-carbon metabolic factors and the risk of RCC.

The study limitations were as follows: (1) the adjusted models differed across the included studies, with variable factors playing an important role in the development of RCC; (2) we could not differentiate the effects of one-carbon metabolic factors from confounding factors including BMI, smoking, alcohol status, and history of hypertension due to limited evidence; (3) publication bias and restricted cubic splines cannot available due to few studies reported the relationship between one-carbon metabolic factors and the risk of RCC; (4) publication bias; and finally (5) pooled data, which restricted a more detailed and comprehensive analysis.

The findings of our study indicated that serum vitamin B2, vitamin B6, vitamin B12, and folic acid supplementation were inversely associated with the risk of RCC. Large prospective cohort studies are needed to verify these associations.

## Supporting Information

S1 Checklist PRISMA Checklist. (DOC)

S1 Table Quality scores of prospective cohort studies using the Newcastle-Ottawa Scale. (DOC)
