# Supplementary material for: One-Carbon Metabolic Factors and Risk of Renal Cell Cancer: A Meta-Analysis
Source: PLoS One. 2015 Oct 29;10(10):e0141762. doi: 10.1371/journal.pone.0141762 (PMC4625965; doi:10.1371/journal.pone.0141762)
Supplement: S1 Table — (DOC) [file pone.0141762.s002.doc]

S1 Table. Quality scores of prospective cohort studies using Newcastle-Ottawa Scale.

| Study | Selection | | | | Comparability | Outcome | | | NOS |
| --- | --- | --- | --- | --- | --- | --- | --- | --- | --- |
|  | Representativeness of the exposed cohort | Selection of the non exposed cohort | Ascertainment  of exposure | Demonstration that outcomes was not present at start of study | Comparability on the basis of the design or analysis | Assessment of outcome | Adequate follow-up duration | Adequate follow-up rate | Overall score |
| IMRCC | 1 | 1 | 1 | 0 | 1 | 1 | 1 | 1 | 7 |
| NLCS | 1 | 1 | 1 | 1 | 2 | 1 | 0 | 1 | 8 |
| EPIC | 1 | 1 | 1 | 1 | 2 | 1 | 1 | 1 | 9 |
| MCCS | 1 | 1 | 1 | 0 | 1 | 1 | 1 | 1 | 7 |
| ATBC | 0 | 1 | 1 | 1 | 2 | 1 | 1 | 1 | 8 |
| NHS | 1 | 1 | 1 | 0 | 2 | 1 | 1 | 1 | 8 |
| HPFS | 1 | 1 | 1 | 0 | 2 | 1 | 1 | 1 | 8 |
